# Parotidectomy using the Harmonic scalpel: ten years of experience at a rural academic health center

**DOI:** 10.1186/s13005-017-0141-5

**Published:** 2017-05-11

**Authors:** Marc A. Polacco, Andrew M. Pintea, Benoit J. Gosselin, Joseph A. Paydarfar

**Affiliations:** 1 Department of Otolaryngology, Dartmouth-Hitchcock Medical Center, Lebanon, NH USA; 2 Dartmouth-Hitchcock Medical Center, One Medical Center Drive, Lebanon, NH 03766 USA

**Keywords:** Parotidectomy, Harmonic, Blood loss, Operative time, Cost

## Abstract

**Background:**

Parotidectomy is one of the most commonly performed procedures by otorhinolaryngologists. Traditionally dissection is performed with a combination of a steel scalpel and bipolar cautery; however, starting in the early 2000s, the Harmonic scalpel has provided an alternative method for dissection and hemostasis. The purpose of this study is to compare operative time, blood loss, complications, and cost between the Harmonic scalpel and steel scalpel plus bipolar cautery for superficial and total parotidectomy.

**Methods:**

Retrospective cohort of patients who underwent superficial or total parotidectomy with the Harmonic or cold steel between 2000 and 2015. Across 255 patients, comparison between operative time, blood loss, complications, and cost was performed.

**Results:**

Superficial parotidectomy was performed on 120 patients with the Harmonic and 54 with steel scalpel. Total parotidectomy was performed on 59 patients using the Harmonic and 22 patients with cold steel. For superficial parotidectomy, the Harmonic reduced operative time (216 ± 42 vs. 234 ± 54 min, *p* = 0.03) and decreased blood loss (28 ± 19 vs. 76 ± 52 mls, p < 0.05). With total parotidectomy the Harmonic decreased operative time (240 ± 42 vs. 288 ± 78 min, *p* = 0.01) and reduced blood loss (38 ± 21 mls vs. 85 ± 55 mls, p < 0.05). There were no differences in complication rates between groups. Harmonic use was associated with surgical cost reduction secondary to reduced operative times.

**Conclusions:**

The Harmonic scalpel decreases blood loss and operating time for superficial and total parotidectomy. Shorter operative times may decrease the overall cost of parotidectomy.

## Background

The incidence of salivary gland neoplasm has been reported to be 1 – 1.4 per 100,000 people annually [1]. Relatively rare, salivary gland tumors account for 5% of all head and neck tumors, the majority of which occur in the parotid gland [2, 3]. Patients who are diagnosed with a parotid gland tumor often undergo parotidectomy. While commonly performed, the procedure is technically challenging and time-consuming as it requires careful dissection of the facial nerve in a region with high vascularity.

The Harmonic scalpel (HS) (Ethicon, Somerville, NJ), an instrument which utilizes ultrasonic vibrations to induce cutting and immediate coagulation of tissue, was introduced in the early 1990s. A low power setting allows for greater hemostasis and slower cutting, while a high power setting offers less hemostasis but faster cutting ability. Since its introduction, the HS has been shown to reduce operative time and intra-operative blood loss across a range of otolaryngologic procedures including thyroidectomy, parotidectomy, glossectomy, and neck dissection [4–7].

The HS reduces bleeding and prevents thermal injury to surrounding tissues greater than 2–3 mm distance, making it an ideal instrument for procedures requiring fine dissection [8, 9]. Prior studies have shown that the HS is useful for reducing blood loss and operative time in superficial and total parotidectomy procedures when compared to using a steel scalpel and bipolar cautery; however, most have contained relatively small cohorts over brief study periods [10–12]. To our knowledge, this is the largest study comparing parotidectomy outcomes between the HS and steel scalpels plus bipolar cautery (SB), and the first to report superficial and total parotidectomy outcomes separately (Table 1). Moreover, this study reports the effect HS use has on the overall cost of performing a parotidectomy.

**Table 1:** Literature comparing parotidectomy outcomes

Surgery	Instrument	No.	OR Time (min)	Blood Loss (ml)	Drain Output (ml)
Superficial parotid					
Muhanna et al. 2014 [12]	SB	32	163.12 ± 21.8	NR^a^	73.5 ± 38.2
	HS	26	137.3 ± 18.6	NR^a^	68 ± 22.3
Blankenship et al. 2004 [11]	SB	21	195.5 ± 37.5	60.0 ± 37.1	48.7 ± 33.8
	HS	19	167.5 ± 42.6	37.5 ± 25.8	48.0 ± 22.7
Jackson et al. 2005 [10]	SB	37	NR^a^	68 ± 12	NR^a^
	HS	35	NR^a^	38 ± 4.23	NR^a^
Polacco et al.	SB	54	234 ± 54	76 ± 52	43 ± 36
	HS	120	216 ± 42	28 ± 19	24 ± 15
Total parotid					
Jackson et al. 2005 [10]	SB	4	NR^a^	NR^a^	NR^a^
	HS	9	NR^a^	NR^a^	NR^a^
Polacco et al.	SB	22	288 ± 42	85 ± 55	33 ± 20
	HS	59	240 ± 78	38 ± 21	35 ± 30
Superficial and Total parotid					
Deganello et al. 2014 [5]	SB	63	151.6 ± 54.1	NR^a^	78 ± 81
	HS	67	146.9 ± 39.9	NR^a^	69 ± 52
Jackson et al. 2005 [10]	SB	41	200.5 ± 41.43	66.0 ± 10.8	NR^a^
	HS	44	183.88 ± 58.17	38.0 ± 3.6	NR^a^

^a^ *NR* not reported

## Methods

**Fig. 1:**
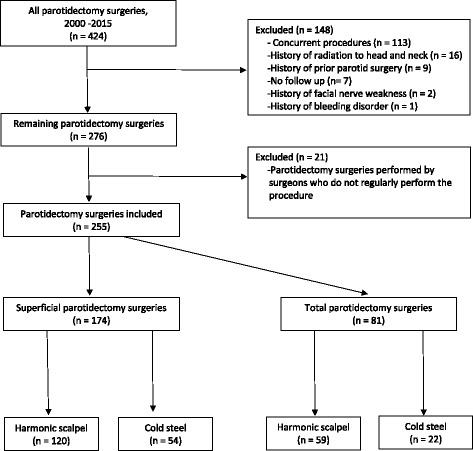
Inclusion criteria flow chart

The medical records of all patients who underwent superficial or total parotidectomy at Dartmouth-Hitchcock Medical Center from 2000–2015 were retrospectively reviewed after gaining approval from the institutional review board. A total of 424 cases were identified. Cases were excluded if the patient had history of prior parotid surgery, radiation, a bleeding disorder, prior facial nerve disorder, was lost in follow up, or if they underwent a combination of procedures such as parotidectomy with neck dissection. This resulted in exclusion of 148 cases. An additional 21 cases were excluded as they were performed by two surgeons who did not routinely perform parotidectomies, defined as less than five parotidectomies per year (Fig. 1).

All included cases were performed by two surgeons (Table 2). For both surgeons, cases prior to 2006 were performed with a combination of steel scalpels and bipolar cautery, while most cases after 2006 were performed with the Harmonic scalpel. A resident surgeon was present in 81% of cases.

**Table 2:** Cases per surgeon

Parotidectomy	Surgeon 1	Surgeon 2
Superficial (SB)^a^	21	33
Superficial (HS)^a^	64	56
Total (SB)^a^	13	9
Total (HS)^a^	28	31
Sum Total Cases	126	129

^a^ *SB* steel scalpel plus bipolar cautery, *HS* Harmonic scalpel

In addition to categorizing cases according to superficial and total parotidectomy with or without use of the HS, cases meeting inclusion criteria were assessed for patient age, sex, operative time, blood loss, post-operative drain output, length of follow up, and complications. Complications assessed included hematoma, seroma, Frey’s syndrome, facial nerve weakness, auricular numbness, keloid, and first bite syndrome. The cost of each procedure was calculated using the reported operating room cost per minute for superficial and total parotidectomy multiplied by total minutes to procedure completion. If the HS was opened, the cost of the instrument was added to the cost of the surgical case. The percentage of cost reduction was calculated by taking the ratio of cost of parotidectomy with HS to that of SB, averaged across all procedures. Statistical analyses were conducted using unpaired t-tests for contiguous data and Fisher’s exact test for categorical data (Microsoft Excel 2013, Redmond, WA).

## Results

### Superficial parotidectomy

A total of 174 patients underwent superficial parotidectomy, 120 with the HS and 54 with SB. There was no significant difference for patient age, sex, and mean follow up duration between groups. Use of the HS compared with SB resulted in shorter duration of surgery (216 ± 42 vs. 234 ± 54 min, *p* = 0.03) and less blood loss (28 ± 19 mls vs. 76 ± 52 mls, p < 0.05), but no significant difference in post-operative drain output (24 ± 15 mls vs. 43 ± 36 mls, *p* = 0.09) or complications (Table 3). Taking the cost of the HS into account, there was a 5.6% average reduction in cost for superficial parotidectomy procedures when the HS was used.

**Table 3:** Patient characteristics and outcomes

	Superficial (*n* = 174)			Total (*n* = 81)		
Variables	HS (*n* = 120)	SB (*n* = 54)	*P* value	HS (*n* = 59)	SB (*n* = 22)	*P* Value
Mean age (years)	59 ± 14	56 ± 15	0.19	56 ± 15	55 ± 14	0.75
Sex						
Male	56 (47%)	24 (43%)	0.87	31 (53%)	10 (45%)	0.62
Female	64 (53%)	30 (56%)		28 (47%)	12 (55%)	
Blood Loss (ml)	28 ± 19	76 ± 52	<0.05	38 ± 21	85 ± 55	< .05
Drain Output (ml)	24 ± 15	43 ± 36	0.09	35 ± 30	33 ± 20	0.78
OR Time (min)	216 ± 42	234 ± 54	0.03	240 ± 42	288 ± 78	0.01
Length of Follow Up (mo)	7 ± 12	7 ± 7	0.13	9 ± 6	13 ± 12	0.17
Complications						
Auricular Numbness	7 (5%)	2 (4%)	0.72	5 (8%)	3 (14%)	0.68
Transient facial paresis	1 (0.8%)	2 (4%)	0.23	1 (1.6%)	1 (4.5%)	1
Permanent facial paresis	0	0	1	1 (1.6%)	0	1
Facial paralysis	0	0	1	0	0	1
Hematoma	0	0	1	0	0	1
Seroma	1 (0.8%)	0	1	0	0	1
Keloid	1 (0.8%)	0	1	0	0	1
Frey’s syndrome	0	0	1	0	1 (4.5%)	1
First Bite	1 (0.8%)	0	1	0	0	1
Average Cost (including cost of Harmonic Scalpel)	$23,190	$24,570		$25,710	$30,240	

### Total parotidectomy

In the total parotidectomy group, there were a total of 81 patients who met inclusion criteria, 59 of whom underwent surgery using the HS and 22 with SB. There was no significant difference in regard to patient age, sex, and mean follow up duration between groups. With total parotidectomy, use of the HS compared with SB resulted in shorter duration of surgery (240 ± 42 vs. 288 ± 78 min, *p* = 0.01) and less blood loss (38 ± 21 mls vs. 85 ± 55 mls, *p* < 0.05), but no significant difference in post-operative drain output (35 ± 30 mls vs. 33 ± 20 mls, *p* = 0.78) or complications. HS use resulted in a 15% average cost reduction of total parotidectomy.

## Discussion

The HS utilizes ultrasonic vibration to denature proteins, forming a coagulum for hemostasis while also limiting thermal injury to surrounding tissue. Since the introduction of the HS, it has been shown to be effective in decreasing operative blood loss across a variety of procedures, from total colectomy to hepatectomy [13, 14]. In the otolaryngology literature, the HS has been shown to decrease blood loss and operative times for thyroidectomies, parotidectomies, and neck dissections [10, 15, 16].

This study is the largest to date comparing the Harmonic Scalpel to cold steel in parotidectomy. Our results corroborate prior parotidectomy studies on the HS, showing both a reduction in blood loss and decrease in operating room time when compared to SB (see Table 1). The benefit of using the HS in total parotidectomy procedures is even more compelling as the differences in blood loss and operative time between groups was greater. While the difference in blood loss between groups in this study was significant statistically, it is unlikely that the volume of blood saved using the HS is clinically significant.

For both the superficial and total parotidectomy groups, there was a significant difference in operative time between the use of the HS and SB dissection. For the superficial group, the amount of time saved using the HS equated to 18 min, while this difference increased to 48 min in the total parotidectomy group. At our institution the amount of operating room time saved, even in the superficial parotidectomy group, translates into a cost reduction greater than the cost of the HS, resulting in a $1381 (5.6%) and a $4530 (15%) decrease in cost of performing superficial and total parotidectomy respectively. We expect the percentage of cost reduction to be relatively consistent across institutions, whereas the monetary value could be highly variable depending on operating room utilization cost per institution. These data are compelling as health-care costs continue to soar in the United States and cost reduction efforts become increasingly important. In 2014, 17.1% of the gross domestic product was allocated for health-care, and the Congressional Budget Office estimates this figure to increase to 25% by 2025 should the rate of increasing expenditures remain constant [17]. Being at the forefront of health-care expenditure, physicians have an obligation to create efforts to control cost in order to continue to provide accessible quality health-care [18].

Moreover, with a decrease in operative time there is also a realized reduction in opportunity cost. Opportunity cost is traditionally defined as the value of a rejected opportunity or alternative [19]. By reducing the overall operative time allocated to performing a parotidectomy, particularly total resections, time resources may be redistributed to endeavors such as additional cases, research, or education. Additionally, this reduction in opportunity cost could translate to increased patient access to providers.

A weakness of this study is that there is no method to determine the degree of resident involvement in the 81% of cases in which there was a resident present. While it could be presumed that junior residents would operate at a slower rate than senior residents, the amount of actual operating time for each resident is likely highly variable as senior residents are granted more autonomy while there is traditionally more attending physician involvement when a junior resident is operating. While one of two attending physicians were present for all cases reported, it is possible that surgeons operating without a resident may not experience a significant difference in blood loss, operating time, or cost.

## Conclusion

Use of the HS for superficial and total parotidectomy is associated with a significantly shorter duration of surgery and less blood loss when compared to use of SB. Shorter operative times were great enough to generate cost savings to offset the cost of the HS and decrease the overall cost of parotidectomy.
